# Dynamics in Morbidity Markers and Cytological Observations Made in Urine of *Schistosoma haematobium*-Infected Children: Pre- and Post-Praziquantel Treatment in an Endemic Setting

**DOI:** 10.3390/medsci10010014

**Published:** 2022-02-17

**Authors:** Patience B. Tetteh-Quarcoo, Asamoah Ampong, Nicholas T. K. D. Dayie, John Ahenkorah, Emilia A. Udofia, Emmanuel Afutu, Robert Aryee, Benjamin P. Niriwa, Fleischer C. N. Kotey, Eric S. Donkor, Patrick F. Ayeh-Kumi

**Affiliations:** 1 Department of Medical Microbiology, University of Ghana Medical School, Accra P.O. Box KB 4236, Ghana; ampong.asamoah@yahoo.com (A.A.); ntkddayie@ug.edu.gh (N.T.K.D.D.); eafutu@ug.edu.gh (E.A.); bobby200055@gmail.com (R.A.); pullebenjamin@gmail.com (B.P.N.); fcnkotey@flerholiferesearch.com (F.C.N.K.); ericsdon@hotmail.com (E.S.D.); pfayeh-kumi@ug.edu.gh (P.F.A.-K.); 2 Department of Anatomy, University of Ghana Medical School, Accra P.O. Box KB 4236, Ghana; 3 Department of Community Health, University of Ghana Medical School, Accra P.O. Box KB 4236, Ghana; eudofia@ug.edu.gh; 4 FleRhoLife Research Consult, Teshie, Accra P.O. Box TS 853, Ghana

**Keywords:** cytology, schistosomiasis, microscopy, *S. haematobium*, morbidity

## Abstract

Background: Schistosomiasis is a neglected tropical disease caused by helminths of the genus *Schistosoma*. Morbidity markers and cytological observations such as squamous metaplastic cells, inflammatory cells, and hyperkeratotic cells in the urine of *S. haematobium*-infected children may suggest disease severity. They may also help predict severe forms of clinical presentation, such as bladder cancer in later years, among infected ones who miss out on early detection and treatment. Insights into possible changes in the morbidity markers and cytological observations in the urine of these *S. haematobium*-infected children before and after treatment would be of high clinical importance. Aim: The aim of this study was to identify changes/dynamics in morbidity markers and cytological abnormalities in the urine deposits of *S. haematobium*-infected children, pre- and post-praziquantel treatment. Methodology: This was a longitudinal study involving baseline and follow-up sampling among basic school children living in schistosomiasis-endemic communities. Urine samples were collected from 520 children at baseline and examined for *S. haematobium* ova by microscopy, while urine chemistry analyses were used for the examination of morbidity markers. The cytological analyses involved cytopathological examination of the urine deposits. Children whose urine showed positivity for *S. haematobium* eggs were treated with a single oral dose of praziquantel (40 mg/kg), after which urine chemistry and cytological analyses were repeated weekly for comparison with baseline, until the eighth week. Results: Morbidity markers such as hematuria, proteinuria, and leukocyturia were detected both at baseline and post-treatment among the infected children (30/520). Hematuria was the predominant parameter (90%, 27/30) detected at baseline, followed by proteinuria (53.3%, 16/30). Leukocyturia was the rarest parameter detected at baseline (13.3%, 4/30). However, almost all these parameters declined gradually post-treatment. Regarding cytological analyses, inflammatory cells were observed most (70.0%, 21/30) at baseline. For hyperkeratotic cells and squamous metaplastic cells, 46.7% and 26.7% were respectively observed at baseline, all of which gradually declined during the weekly follow-ups. Notably, squamous metaplastic cells persisted in all the participants from Week 1 through Week 3 post-treatment, but declined gradually thereafter. Conclusions: Morbidity markers and cytological observations in the children gradually decreased after treatment. Therefore, we continue to recommend routine cytological screening for urogenital schistosomiasis patients at hospitals in *S. haematobium*-endemic locations using both baseline and follow-up samples to detect these abnormalities early and monitor changes that may be occurring after treatment. Such changes may be useful in assessing treatment progress in infected persons.

## 1. Introduction

Urogenital schistosomiasis is a parasitic disease caused by *Schistosoma haematobium* (*S. haematobium*). It mainly manifests itself as passage of blood in urine, also known as hematuria, and is increasingly becoming a public health problem. In endemic communities in Ghana, there is a high prevalence of hematuria due to *S. haematobium* infection among school-aged children [1,2]. Even though urban schistosomiasis has emerged over the years, the disease mostly occurs in rural communities within which water bodies such as lakes, ponds, and streams can be found [3,4]. Among populations at risk for *Schistosoma* infection are children and others who may use infected water for either domestic purposes or as a means of recreation [5].

Currently, the drug of choice for the treatment of schistosomiasis is praziquantel (PZQ), which has been at the core of schistosomiasis control strategies in many countries [6,7]. So far, it is highly effective against all schistosome species that infect humans, even though there have been some cases of possible resistance or persistent infections [8,9,10,11].

Some studies have linked squamous cell bladder cancer with persistent infection with *S. haematobium* [1,12,13]. In considering a routine noninvasive diagnostic procedure used to detect cancer of the urinary tract (primarily, bladder cancer), cytopathological examination of urine samples has been recommended [14,15]. During the follow-up procedures of patients previously treated for bladder cancer, cytopathological examinations have been used to detect recurrence of the cancer, as early as possible [15]. In high-risk populations, cytopathological examination of urine is predominantly used for the screening of urothelial carcinoma [14]. Cystoscopy (which is considered invasive as compared to cytopathological examination of urine) can also be used to investigate symptoms that involve the urethra or the bladder [15]. In cystoscopy, a thin tube (cystoscope) is inserted into the urinary opening, threaded through the urethra and into the bladder. A small camera attached to the cystoscope relays live video feed to a monitor, which can be viewed by the healthcare provider [15]. It is, therefore, not surprising that cytopathological examination of urine samples has been recommended for the screening of cancer of the urinary tract (primarily, bladder cancer) [14,15]. Previously, we presented findings from cytological and wet-mount microscopic observations made in the urine of *S. haematobium*-infected children living in endemic communities in the capital city of Ghana (the southern belt) [1]. Cytopathological examination of the urine deposits was a key technique used in that study [1]. In the said study, we demonstrated the presence of squamous metaplastic cells, inflammatory cells, hyperkeratotic cells, and urothelial/transitional cells in the urine samples examined [1]. It was concluded that some of these observations (squamous cell metaplasia, inflammatory cells, and hyperkeratosis) made in the *S. haematobium*-infected children may play an important role in the disease condition, leading to a severe form of clinical presentation, such as bladder cancer in later years, if early attention is not given [1]. Such attention may include early treatment; thus, it would be interesting to know what observations could be made in *S. haematobium*-infected children who are treated as soon as they are diagnosed, with weekly follow-ups—a gap the current study sought to fill. The aim of this study was, therefore, to identify changes/dynamics in morbidity markers and cytological observations in the urine deposits of *S. haematobium*-infected children in the middle belt of Ghana (a location different from that of our earlier study), pre- and post-praziquantel treatment.

## 2. Materials and Methods

### 2.1. Study Design and Study Sites

This was a longitudinal study involving baseline and follow-up sampling among 520 basic school children living in schistosomiasis-endemic communities along the stretch of the Volta Lake within the Pru District of the then Brong-Ahafo Region of Ghana (currently the Bono East Region), which is in the middle belt of Ghana. People within these communities use the river for fishing, farming, washing, and recreational activities. The district is one of the areas in Ghana with a high prevalence of schistosomiasis, recording an above 50% *S. haematobium* infection rate in 2010 [5], making it a suitable site for the current study.

Questionnaires were administered to gather demographic (age, water source, etc.) and clinical (visible hematuria, intake of anti-helminthics, especially praziquantel, and others) information, after informed consent from parents, guardians, teachers and assent from the children had been obtained. This information aided in identifying children who met the inclusion criteria, such as visiting the water source (lake) and not taking anti-helminthic medication (for schistosomiasis) prior to sample collection.

Clean, dry, screw-capped, wide-mouthed, and leak-proof urine containers were given out to the selected participants, each of whom returned with about 20 mL of urine after being carefully instructed on how to collect the samples. The samples were immediately screened for morbidity markers by urine chemistry analysis and *S. haematobium* ova by microscopic identification of its characteristic ovoid shape with a terminal spine. It is noteworthy that, for the purpose of this study, identification of the ova did not need the use of molecular tests; therefore, it would not have been possible to differentiate *S. haematobium* from its hybrids, such as *S. haematobium–S. bovis* hybrids, just by using microscopy.

The positive samples were subsequently transported to the parasitology laboratory of the Department of Medical Microbiology, University of Ghana Medical School (UGMS), Korle Bu, Accra, Ghana, for positivity confirmation and cytological analyses.

Children with positive *S. haematobium* eggs were treated with a single oral dose of PZQ (40 mg/kg), after which urine chemistry and cytological analyses were repeated weekly for comparison with baseline, until the eighth week.

### 2.2. Examination of Morbidity Markers and Detection of S. haematobium Ova

Urine chemistry analysis by dipstick (which is a qualitative assay) was used for the examination of morbidity markers. The collected urine samples were analyzed for hematuria and proteinuria, alongside other urine chemistry parameters (pH, specific gravity, glucose, ketones, bilirubin, urobilinogen, and leucocytes) using a urine chemistry dipstick (URIT 10 *V*, URIT Medical Electronic Co. Ltd., Nanshan District, Shenzhen, China) [16]. The urine chemistry examination was done on baseline and post-treatment samples on a weekly basis until the eighth week.

Wet-mount examination was used for the detection of *S. haematobium* ova. Briefly, 10 mL of each urine sample was centrifuged in a centrifuge tube for 5 min at 5000 rpm speed, to allow for sedimentation of the ova. The supernatant was discarded, and its resultant leftover was used in the resuspension of the sediment to make a total volume of 0.5 mL. Fifty microliters of the resuspended urine sediment was pipetted onto a glass microscope slide (Biogenix Inc Private Ltd., Lucknow, Uttar Pradesh, India), and a cover glass placed on it, to examine for *S. haematobium* ova using the ×10 and ×40 objectives of an optical light microscope (Leica Galen III, catalogue E no. 317506, serial no. ZG6JA4).

### 2.3. Cytological Examination of Urine Deposit

About 4 mL of each urine sample was centrifuged at a speed of 1500 rpm for 10 min. The supernatant was poured away, and the resultant leftover (1 mL) was used in the resuspension of the sediment, similar to what was done in the case of the ova detection. Approximately 3–5 drops of the re-suspended deposit was used to make a smear on a microscope slide by cytocentrifugation, to prepare a monolayer smear of urine deposits directly onto the slide [17]. The smear slides were wet-fixed with 95% ethyl alcohol for a minimum of 15 min, before staining using the Papanicolaou staining method that employs hematoxylin, Orange G, and eosin alcohol stains [18]. The stained slides were examined microscopically for cytological abnormalities, such as squamous cell metaplasia, inflammation, and hyperkeratosis.

The cytological examination was done on baseline and post-treatment samples on a weekly basis up to the eighth week to identify cytological changes/dynamics in the urine deposits of *S. haematobium*-infected children, weeks after treatment.

### 2.4. Statistical Analysis

Data obtained were stored in Microsoft Excel and analyzed using the Statistical Products and Services Solutions (IBM^®^ SPSS^®^ version 25.0). Data were summarized by determining frequencies of morbidity markers and cytological observations in the urine deposits, as well as a comparison of weekly observations.

### 2.5. Ethical Statement

This work was approved by the Ethical and Protocol Review Committee of the College of Health Sciences, University of Ghana, Accra, Ghana (Protocol ID no: CHS-Et/M.8—P.2.11/2017–2018). Informed consent and assent were obtained from teachers, parents, guardians, and school children before sampling.

## 3. Results

### 3.1. S. haematobium Detection and Morphological Forms

**Figure 1:**
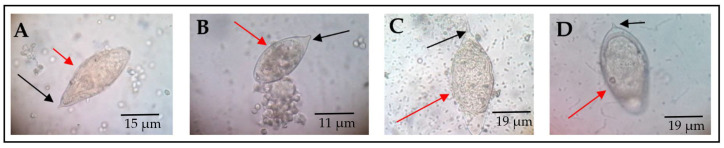
Morphological forms of *S. haematobium* ova. The red arrows point to the eggs, while black arrows indicate the spine. All eggs represented by (**A**–**D**) have the usual characteristic oval shape and terminal spine. However, (**B**) has a blunt spine, while (**D**) appears to be oval to round-shaped, with a slightly curved spine. These pictures were chosen from different participants as representative observations (source of images: authors’ laboratory work).

Out of the 520 participants enrolled, 34 were found with *S. haematobium* infection, representing a 6.5% infection rate. Morphological features in terms of shape and spine position of *S. haematobium* ova were observed (Figure 1).

### 3.2. Clinical Morbidity Markers Detected among S. haematobium-Infected Children

**Figure 2:**
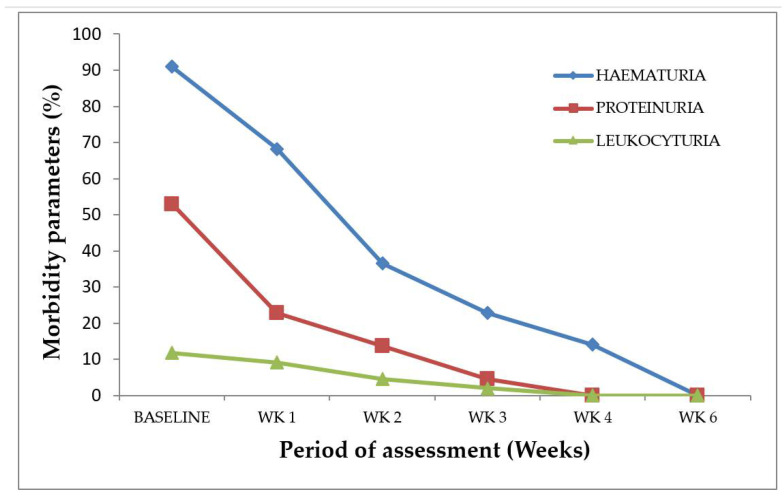
*S. haematobium* infection morbidity indicators at baseline and post-treatment. *n* = 30, WK represents week.

Morbidity markers usually associated with *S. haematobium* infection, such as hematuria, proteinuria, and leukocyturia (presence of pus cells in urine) were detected both at baseline and post-treatment among the infected study participants. Four participants were lost during the follow-ups and, thus, were excluded from the analyses. Therefore, hematuria was the predominant parameter (90%, 27/30) detected among the 30 *S. haematobium*-infected participants at baseline (Figure 2). However, there was a gradual decline in hematuria among the post-treatment study participants—66.7% (20/30) at Week 1, 36.7% (11/30) at Week 2, 26.7% (8/30) at Week 3, and 16.7% (5/30) at Week 4. With regard to proteinuria, 53.3% (16/30) was detected at baseline, and 26.7% (8/30), 13.3% (4/30), and 3.3% (1/30) were respectively recorded during follow-up at Week 1, Week 2, and Week 3, whilst, at Week 4, no form of proteinuria was detected (Figure 2). Leukocyturia was the rarest parameter detected among the infected participants. The percentage leukocyturia at baseline was 13.3% (4/30); this proportion declined to 10.0% (3/30) and 6.7% (2/30) during the follow-ups at Week 1 and Week 2, respectively, while Week 3 had 3.3% (1/30) and Week 4 recorded no leukocyturia (Figure 2). Proteinuria, hematuria, and pyuria were not detected during the sixth week of the post-treatment assessment (Figure 2).

### 3.3. Cytological Observations Made in the Urine

**Figure 3:**
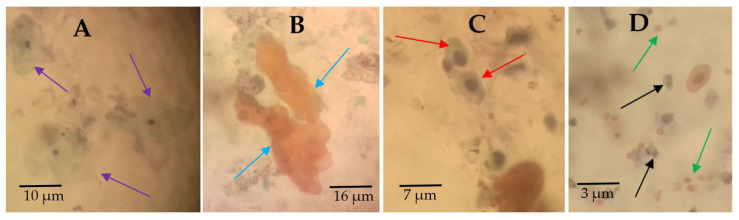
Urine cytology showing normal and abnormal urothelial cells. Papanicolaou-stained urine deposit smears showing (**A**) arrows pointing at normal urothelial cells from noninfected participants, (**B**) arrows pointing at anucleated hyperkeratotic clustered cells, (**C**) arrows pointing at squamous metaplastic cells, and (**D**) black arrows pointing at inflammatory cells and green arrows pointing at red blood cells. These pictures were chosen from different participants as representative observations (source of images: authors’ laboratory work).

Various cytological observations were made in the Papanicolaou staining of the urine smears (Figure 3). The main cytological observations made in the samples were hyperkeratotic cells (Figure 3B), squamous metaplastic cells (Figure 3C), and inflammatory cells (Figure 3D).

### 3.4. Cytological Abnormalities at Baseline

Generally, inflammatory cells were observed in most (70.0%) of the urine samples from the *S. haematobium*-positive cases subjected to the cytological analysis (Table 1). Hyperkeratotic cells and squamous metaplastic cells were observed in 46.7% and 26.7% of the *S. haematobium* positive samples, respectively (Table 1). Red blood cells were found in 60.0% of the *S. haematobium*-positive cases by cytological analysis.

### 3.5. Cytological Changes in the Post-Treatment Weekly Follow-Ups

**Figure 4:**
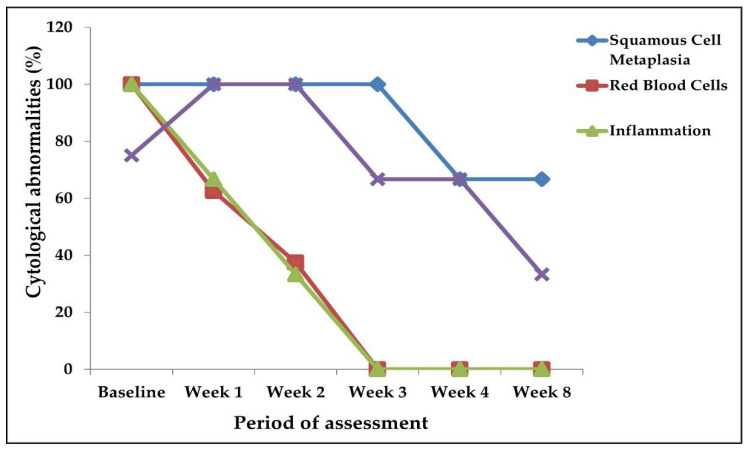
Cytological changes at baseline and post-treatment.

There were improvements in the cytological observations made during the post-treatment urine cytology from Week 1 up to the eighth week, compared to the baseline (Figure 4). Squamous cell metaplasia was persistent (100%, 8/8) in the participants who had it at baseline, from Week 1 to Week 3, post-praziquantel treatment (Figure 4). However, at Week 4 and Week 8, squamous cell metaplasia was found in 62.5% (5/8) apiece of those cases (Figure 3). Inflammation reduced from 100% (21/21) at baseline to 61.9% (13/21) and 38.1% (8/21) at Weeks 1 and 2, respectively, whilst, at Week 3, Week 4, and Week 8, no inflammatory cells were found in each case (Figure 4). The occurrence of hyperkeratosis also persisted from baseline to Week 1 and Week 2 (100%, 14/14). It, however, reduced to 64.3% (9/14) at Week 3 and Week 4, and further reduced to 35.7% (5/14) at the Week 8 post-treatment assessment (Figure 3). From the baseline (*n* = 18) (Table 1), Week 1 and Week 2 had red blood cells persisting in 61.1% (11/18) and 38.8% (7/18) of the cases, respectively (Figure 4). However, there were no red blood cells at Week 3, Week 4, and Week 8 post-treatment (Figure 4).

### 3.6. Case Representation of Observation at Baseline and Reduction in Cytological Abnormalities in the Weekly Post-Treatment Analysis

**Figure 5:**
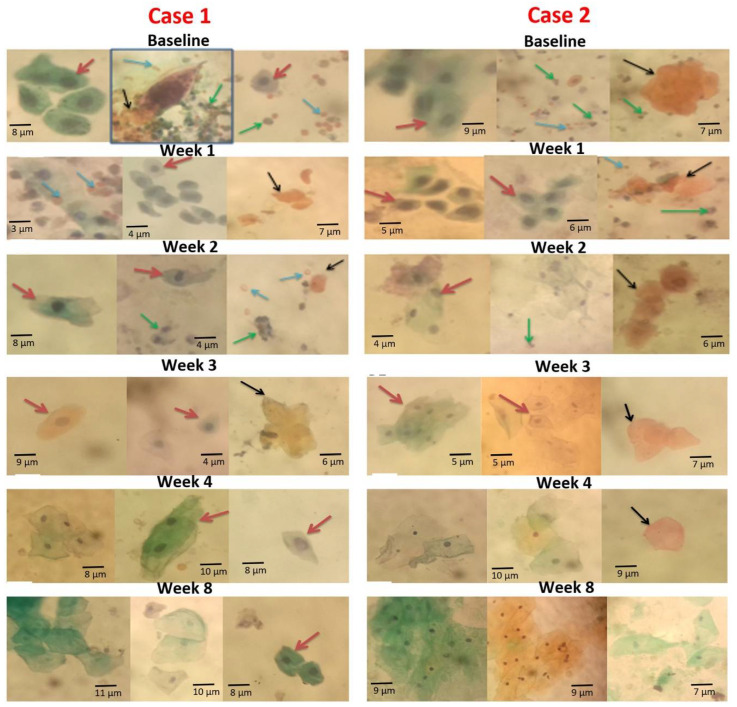
Papanicolaou-stained smear showing urothelial cell changes at baseline and post-PZQ treatment for Cases 1 and 2. Red arrows indicate squamous metaplastic cells, black arrows indicate hyperkeratotic cells, blue arrows indicate red blood cells, and green arrows indicate inflammatory cells. Baseline, Week 1, Week 2, Week 3, Week 4, and Week 8 indicates each time point for Case 1 and Case 2. All images in Case 1 are from one participant, whiles images in Case 2 are from another participant (source of images: authors’ laboratory work).

A pictorial view of the smears from two cases (Cases 1 and 2) is presented in Figure 5 to show the occurrence of cytological changes (dynamics) at baseline and weekly post-praziquantel treatment follow-ups. Generally, there were more urothelial cells excreted in the urine of *S. haematobium*-infected participants at baseline, with more abnormalities (Figure 5). Squamous metaplastic cells, hyperkeratotic cells, inflammatory cells, and red blood cells markedly occurred in both cases at baseline (Figure 5). These observations made at baseline were not different from that of Week 1 post-treatment, since all the abnormalities were still seen at this stage in the smears for both cases. At post-treatment Week 2, both hyperkeratotic and squamous metaplastic cells persisted in both cases, but Case 1 recorded more squamous metaplastic cells than did Case 2. A few red blood cells and inflammatory cells occurred at Week 2 (Figure 5). Week 3, Week 4, and Week 8 revealed no inflammatory cells and red blood cells for both cases. Hyperkeratotic cells were scarce at Week 3, Week 4, and Week 8, for the smears in both cases (Figure 5). There were a few squamous metaplastic cells at Week 3 for both cases, with some nuclear enlargements (Figure 5). At Week 4, there were no squamous metaplastic cells in Case 2, but Case 1 still had a few. The Week 8 assessment found most cells with normal morphology in both cases, but a few isolated ones in Case 1 (Figure 5).

## 4. Discussion

This study aimed at identifying changes/dynamics in morbidity markers and cytological observations made in the urine deposits of *S. haematobium*-infected children (in the middle belt of Ghana) pre- and post-praziquantel treatment. Previously, we presented findings from cytological and wet-mount microscopic observations made in the urine of *Schistosoma haematobium*-infected children living in endemic communities in the capital city (southern belt) of Ghana [1]. In that study, we reported that some of the observations (squamous cell metaplasia, inflammatory cells, and hyperkeratosis) made in the *S. haematobium*-infected children may be critical in understanding the severity of the disease, such as bladder cancer development in later years, in the absence of early medical attention [1]. It was, therefore, necessary that this study was conducted to provide insights into any possible changes that could occur in cytological observations and morbidity markers among *S. haematobium*-infected children, before and after treatment.

The detection of hematuria, proteinuria, and leukocyturia at baseline, which gradually reduced per weekly analysis after praziquantel treatment in this study, agrees with findings of previous studies [11,19,20]. In a different location in Ghana, we earlier reported a hematuria incidence of 56% using urine reagent strips among children [1]. Therefore, the 90% occurrence of hematuria at baseline observed in this study can be considered relatively high. The high occurrence of hematuria (90%) and proteinuria (53.3%) due to *S. haematobium* detected with the urine test strip at baseline is in congruence with similar studies by Tetteh-Quarcoo et al. [11] and Wilkins et al. [21], as well as another related study which asserted that hematuria and proteinuria are associated with urogenital schistosomiasis [22].

The occurrence of these morbidity markers is expected as various researchers have tried to explain the reasons for detecting these markers in the urine [20,21,22,23,24]. With hematuria, the attachment of the spine of *S. haematobium* ova to the inner walls of the bladder have been said to leave some lesions, leading to the presence of small bleeds or the presence of blood in the urine of the infected participants, even after termination of egg output [20,22]. On the other hand, proteinuria has been attributed to glomerular damage due to infections and immune complexes [23]. Other researchers have, however, associated protein in the urine with intensity of infection, suggesting that the protein is likely to originate from the lesions in the bladder and ureter created by the deposition of *S. haematobium* eggs [21,24].

Hematuria, proteinuria, and leukocyturia have been reported to decrease after treatment with praziquantel [5,11,20]. In the current study, the weekly reduction in the occurrence of hematuria, proteinuria, and leukocyturia, which persisted up to the third week after treatment agrees with the findings by Wami et al. [25] and Tetteh-Quarcoo et al. [11], who attributed the reduction in these clinical morbidity indicators to the gradual healing of lesions and wounds created by eggs deposited in the walls of the bladder. Furthermore, the outcome of the post-treatment analysis in this study is in accordance with a similar study conducted by Stete et al. [20] in which the authors asserted that reductions in morbidity indicators (hematuria, proteinuria, and leukocyturia) serve as additional parameters for assessment of praziquantel efficacy. Thus, observation of these patterns of morbidity markers after treatment may be useful in assessing treatment progress in infected persons.

Cytological observations such as squamous cell metaplasia, hyperkeratosis, and inflammation found in this survey among participants infected with *S. haematobium* suggest a possible link between urothelial cell changes and urogenital schistosomiasis. Similar studies conducted in Nigeria and Kenya related *S. haematobium* infection with cytological abnormalities, such as severely dysplastic squamous cells and urinary tract hyperplasia, which are precursors of bladder cancer [12,26].

In this study, the observation of inflammation followed by hyperkeratosis and then squamous cell metaplasia at baseline is similar to our earlier study among Ghanaians in a different geographical location—the southern belt), where inflammatory cells were the cytological abnormalities in the majority of the samples, followed by hyperkeratosis and squamous cell metaplasia [1]. An earlier study in an *S. haematobium*-endemic Msambweni area of Kwale County, located in southeastern Kenya, formerly in Kwale District of Coast Province [26], made similar observations; the prevalence of inflammation was the highest (39%), followed by metaplasia (33%), hyperkeratosis (30%), and frank atypia (0.4%). Metaplastic squamous cells could be as a result of chronic inflammation of the urothelium in response to stimulating factors such as *S. haematobium* ova trapped in the bladder tissues [27]. This indicates the relationship between inflammation and development of squamous cell metaplasia [26].

In Ghana, a study conducted on tissue schistosomiasis at the Korle-Bu Teaching Hospital, Accra, Ghana (located in the southern part of the country), by Der et al. [28] found a possible link between *S. haematobium* infection and squamous cell carcinoma. This was a retrospective study conducted from 2004 to 2011, in which 98.7% of the tissue schistosomiasis cases were attributed to *S. haematobium* infection [28]. The authors noted that in 93.6% of cases, the affected organ (having malignant squamous cell abnormalities) was the urinary bladder [28].

Cytological changes observed during the first 3 weeks to Week 8 post-treatment, relative to the baseline, suggests that some cytological abnormalities can resolve with time among these children, and this agrees with the thought that transitional metaplastic squamous cells can revert to normal forms several weeks after treatment [29]. Studies have shown that squamous cell metaplasia is reversible; however, persistent exposure to factors that cause metaplasia may induce malignant transformation and development of cancer [29]. The observance of a reduction in inflammatory cells after treatment agrees with the assertion that continuous egg deposition in the urothelium is linked to chronic inflammation, leading to squamous cell abnormalities and development of fibrosis [30]. Keratinizing cells with inflammation have been found to have a strong association with cancer development [26]. Keratinization usually occurs in response to changes such as pressure and other forms of local irritation within the cellular environment [31].

Detection of hematuria both at baseline and after praziquantel treatment with a urine dipstick test strip and urine cytology (Papanicolaou staining) in the current study is in line with previous findings suggesting hematuria as an indicator for diagnosing *S. haematobium* infection and assessing the chemotherapeutic effect after treatment with praziquantel [11,21,32]. While the results of this study confirm that both the dipstick and the Papanicolaou stain showed complementary results in detecting hematuria, the seemingly higher sensitivity of the dipstick to detect hematuria over the Papanicolaou-stained microscopy (also observed in the post-treatment weekly follow-ups) could be attributed to the ability of the dipstick to detect both hemolyzed and intact red blood cells, while the Papanicolaou stain mainly shows intact cells, and not lysed cells [33,34].

Some important deductions that could be made from the current study, which could have a direct impact on clinical practice, are as follows: (1) cytological abnormalities observed should not be expected to vanish/disappear within days of praziquantel treatment but weeks (even up to 8 weeks); (2) there is no need to be too anxious regarding ‘instant’ progression to squamous cell carcinomas, since, in the majority of the cases in this study, even serious cytological abnormalities happened to resolve within a couple of months (around 8 weeks) after treatment; (3) it appears that, in the current study, there were no more red blood cells or inflammatory cells detected after the third week post-treatment, which is good news, but also calls for extra attention to be given to those whose samples may persistently exhibit these cells several months after the third week post-treatment. We, therefore, assume that the prognosis for these children in the current study seems fairly good, but some critical questions that need to be answered are as follows: (1) Would this be the picture irrespective of how long or how frequently/recurrently these children were infected? (2) Would it matter if these were elderly people who had been exposed to this infection persistently or in the past when they were younger? (3) Would there be a difference in what we would see if this study was conducted in a totally different geographical area? These are but a few questions that need to be addressed in subsequent studies/research.

## 5. Conclusions

This study observed hematuria, proteinuria, and leukocyturia among *S. haematobium*-infected children at baseline, which gradually reduced per weekly analysis after praziquantel treatment. Similar observations were made in the cytological analyses, where squamous cell metaplasia, hyperkeratosis, and inflammation were found with *S. haematobium* infection at baseline, but also declined in post-treatment samples. Notably, cytological changes observed during the first 3–8 weeks post-treatment, relative to the baseline, indicate that cytological abnormalities in these children can resolve over time. However, it is difficult to know what the situation would be with a different geographical area, age group, or time/frequency of exposure. Therefore, we continue to recommend that routine cytological screening for urogenital schistosomiasis patients at hospitals in *S. haematobium*-endemic locations is conducted on both baseline and follow-up samples to promptly detect these abnormalities and monitor changes that may be occurring after treatment. Additionally, we suggest that both changes/dynamics in cytological observations and morbidity markers after treatment may be useful in assessing treatment progress in infected persons.

## Figures and Tables

**Table 1:** Cytological observations revealed by urine cytology at baseline (*n* = 30).

Cytological Abnormality	*n* (%)
Squamous metaplastic cells	8 (26.7%)
Inflammatory cells	21 (70.0%)
Hyperkeratotic cells	14 (46.7%)
Red blood cells	18 (60.0%)

## Data Availability

All data supporting the reported results have been included in the paper.
